# Household knowledge, practice and treatment seeking behaviors towards cutaneous leishmaniasis in the endemic rural communities of Ganta- afeshum district, Tigrai, northern Ethiopia, 2019: a cross-sectional study

**DOI:** 10.1186/s40794-021-00144-4

**Published:** 2021-06-15

**Authors:** Kebede Tesfay, Fitsum Mardu, Brhane Berhe, Hadush Negash, Haftom Legese, Gebre Adhanom, Getachew Belay, Aderajew Gebrewahid, Shinesh Tesfay, Zenawi Hagos Gufue

**Affiliations:** 1 Unit of Medical Parasitology and Entomology, Department of Medical Laboratory Sciences, College of Medicine and Health Sciences, Adigrat University, Adigrat, Ethiopia; 2 Unit of Microbiology and Immunology, Department of Medical Laboratory Sciences, College of Medicine and Health Sciences, Adigrat University, Adigrat, Ethiopia; 3 Unit of Clinical Chemistry, Department of Medical Laboratory Sciences, College of Medicine and Health Sciences, Adigrat University, Adigrat, Ethiopia; 4 Tigrai Health Research institution, Tigrai, Ethiopia; 5 Department of Public Health, College of Medicine and Health Sciences, Adigrat University, Adigrat, Ethiopia

**Keywords:** Cutaneous leishmaniasis, Ganta-afeshum, Ethiopia, Knowledge, Practice, Treatment-seeking behavior

## Abstract

**Background:**

Cutaneous leishmaniasis is endemic to Ethiopia. However, the prevention and control efforts of leishmaniasis remain unfocused with clear knowledge and practice gaps within the country. Thus, a house to house survey has been carried out to assess the knowledge, practice and treatment-seeking behavior of households towards cutaneous leishmaniasis in the rural communities of Tigrai region, northern Ethiopia.

**Methods:**

A community-based cross-sectional house-to-house survey was conducted in two selected rural villages of Ganta-afeshum district, Tigrai, northern Ethiopia in 2019. A simple random sampling technique was employed to select the participants. Household heads were interviewed using a pre-tested semi-structured questionnaire. Epi info version 7.0 was used for data entry and the data were imported to SPSS version 23 for analysis. Chi-square test (χ2) was used to test the association between the independent variables and the knowledge and practice status of the study participants. *P*-value < 0.05 was used to declare a statistically significant association among the variables.

**Results:**

In our study, most of the participants (78%) stated that cutaneous leishmaniasis is a health problem in the area. Three hundred eighty (99.5%) participants responded that the most common clinical presentation of cutaneous leishmaniasis is a lesion on the face. All of the study participants did not know the mode of cutaneous leishmaniasis transmission, and had never heard of the sand fly. A majority of the participants were unaware of the main prevention methods for cutaneous leishmaniasis. Lastly, traditional medicine was used in 90% of the study households with a previous history for cutaneous leishmaniasis.

**Conclusion:**

There is a lack of awareness regarding the transmission of cutaneous leishmaniasis in Ganta-afeshum, Ethiopia, where the majority of individuals are unfamiliar with the sand fly vector. Prevention methods for cutaneous leishmaniasis were unavailable among the community. Therefore, health education programs concerning cutaneous leishmaniasis transmission, prevention, and treatment in the area should be rigorously implemented.

**Supplementary Information:**

The online version contains supplementary material available at 10.1186/s40794-021-00144-4.

## Background

Cutaneous leishmaniasis (CL) is a serious public health problem throughout the globe. It can manifest as local cutaneous leishmaniasis (LCL), diffuse cutaneous leishmaniasis (DCL), or Mucocutaneous leishmaniasis (MCL) [1]. Globally, about 0.7 to 1.2 million new cases of CL are estimated each year [2]. Cutaneous leishmaniasis is common throughout Africa, South America, Asia, the Middle East, and the Mediterranean regions. The countries Afghanistan, Algeria, Colombia, Brazil, Iran, Syria, Ethiopia, North Sudan, Costa Rica, and Peru account for 70 to 75% of the incidences of CL [3].

In Ethiopia CL is an incredibly neglected tropical disease which significantly affects children between the ages of 10–15 years old [4]. It is the most common form of leishmaniasis, mostly affecting the poorest communities of the world [5, 6]. Annually, an estimated 20,000 to 50,000 new cases of CL have been reported in Ethiopia including, 300 CL cases reported in Silti district, Oromia region [7, 8]. *Leishmania aethiopica* is a main causative agent of CL in Ethiopia; however rare cases due to *Leishmania tropica* and *Leishmania major* have also been reported [9]. The vectors *phlebotomus longipes* and *plebotomus pedifer* most commonly transmit CL in Ethiopia [10]. *Leishmania aethiopica* is predominantly transmitted in a pre-domestic habitat close to rock hyraxes (*Procavia capensis* and *Heterohyrax brucei*), which act as a main reservoir [11, 12]. Areas at risk of CL in the country are those found at high altitudes and with heavy rainfall [13].

Scars of CL on the exposed body parts cause long-term psychological and social consequences. Chronic lesions lead to social stigmatization including ostracism, impaired education, and economic loss. The social consequences of CL particularly affect young women who face marriage difficulties due to post-infection scars [14, 15]. In Ethiopia there has been a limited effort to control and prevent CL [4], due to challenges including poor access to health care, large population movements, low practice of insecticide-treated nets (ITNs), and limited financial and human resources [16]. There has also been poor progress in advancing treatment and diagnosis activities. As few health facilities provide treatment for CL, many affected individuals commonly undergo traditional medicine practices [17, 18].

Despite epidemiological studies previously reported, an information gap surrounding the knowledge, practice, and treatment-seeking behavior of communities towards CL in Ethiopia still remains. There is also a lack of community awareness on the prevention and control methods of CL [11]. Having such information may have an impact for initiating health education programs to minimize the risk of CL in the area. Therefore, in the present study we assessed the knowledge, practice and treatment-seeking behavior of households towards CL in the rural communities of Ganta-afeshum district, Tigrai, northern Ethiopia.

## Materials and methods

### Study design and setting

A community-based cross-sectional study was employed in the selected villages (Mugulat and Bahra-sheheta) of Ganta-afeshum district, Tigrai national regional state, Northern Ethiopia between January and September 2019. Ganta-afeshum is one of the districts of Tigrai regional state, located 905 kms from the capital city of Ethiopia, Addis Ababa. A total of 99,290 individuals live in Ganta-afeshum district, of which 46,826 are male and 52,464 female. More than 85% of the population lives in rural communities. During the study time, there were about 21,587 households in this district, with an average of 4.59 persons per household. The total area of the district is 53,035.30 ha, of which 10,800.4 ha is cultivatable. The climate of Ganta-afeshum is Dega (highland) and Weina Dega (semi-highland), with an altitude of 1800-3500 m above sea level. The annual average rainfall ranges from 140 to 672 mm and the average annual temperature is 18c (6-30C) [19].

### Sample size and sampling procedure

The study district was selected based on previous information from the laboratory logbook in Ayder Comprehensive and Specialized Hospital Dermatology Center, as a significant number of patients with suspected CL were found at these establishments during the assessment. A previous survey conducted in Tigrai region also revealed that CL is a serious public health problem in the area [20]. Two sub-districts (Mugulat and Bahra-Siheta) were selected to assess the knowledge, practice, and treatment- seeking behavior among the selected households in the district.

Due to a lack of similar studies in the area, it is estimated that 50% of the community would have prior knowledge and practice of CL. Therefore, the sample size was calculated with a single population proportion formula with 95% confidence intervals (CI) and 5% margin of error. As a result, a total of 384 household heads aged 18 years old or above participated in the study. A list of households from each sub-district was available in each respective health center or health post. During the study, Mugulat and Bahra-Siheta sub-districts had 1212 and 1335 households, respectively. The total sample size was proportionally allocated to study sub-districts, 183 to Mugulat and 201 to Bahra-Siheta. Finally the study households were selected randomly.

### Data collection

Trained health extension workers of the study area collected all the required data from the household heads during the survey. Socio-demographic data (age, sex, occupation, education level, family size, and distance to health facility) was carefully recorded using a semi-structure questionnaire. The questionnaires were prepared in the native language of the participants (Tigrigna) and adopted from previous similar studies [21–23]. Additionally, the questionnaire was pretested to assess its validity and feasibility on household heads who were not part of the study population but had similar characteristics. The questionnaire is included as a supporting file. Knowledge, practice and treatment seeking behavior of CL from each study household were collected during the house-to-house visits.

### Measurement of knowledge and practice of participants

The knowledge and practice of the respondents to CL were evaluated using different questions. Knowledge of the respondents was assessed using ten-item questions: 1. Have you ever heard about CL? 2. Do you believe that CL is a health problem? 3. Do you believe that CL can be cured through treatment? 4. What do you think the mode of CL transmission? 5. What are the symptoms of CL? 6. Location of lesion or scar? 7. Have you ever heard about the sand-fly? 8. Where does a sand fly breed? 9. Do you believe CL can be prevented? 10. What are the prevention measures of CL? Practice of the households on the prevention and control of CL was evaluated using seven questions: 1. Health education for CL? 2. Did you sleep under bed net to prevent sand fly biting? 3. Was your house sprayed with chemicals to protect sand fly biting in the last 12 months? 4. Control and fill cracks and animal borrow? 5. Do you sleep outdoor during night? 6. Sleeping conditions? 7. Working outsides during night?

Respondents who replied the right answer for each requested question were given a score of 1 and those who responded incorrectly were given a score of 0. The average score of all respective questions was calculated. Hence, individuals with an average score greater than or equal to the mean score were considered to be knowledgeable. The practice of the respondents was similarly evaluated based on the mean score of the respective questions.

### Data analysis

The collected data were coded and checked for clarity, consistency, and completeness up to the end of the data collection period and entered into Epi info version 7.0. The data were imported to SPSS version 23 for analysis. Descriptive statistics of numeric variables are presented in median and inter-quartile range (IQR). Categorical variables are presented using frequencies and percentages. For two by two tables Chi-square test with continuity correction was used to assess associations between the outcome variable and independent variable. Pearson Chi-square test was used to test an association between independent variables having more than two categories, and the dependent variables. The existence of statistically significant associations was considered when the *P*-value was less than 0.05 at 95% confidence level. The result of the demographic characteristics, knowledge, practice, and treatment-seeking behavior of the participants was presented using frequency tables.

## Result

### Socio demographic characteristics of study participants

A total of 384 household heads were surveyed from the selected villages of the district. Out of the total participants, 238 (62%) were female and 146 (38%) were male. The median age of the participants was 38 years (IQR 30–50). Around half 192 (50%) of the study participants were farmers. Regarding education, 56% of the study participants were illiterate (Table 1).

**Table 1:** Socio-demographic characteristics of study participants in Ganta Afeshum district, eastern Tigrai, northern Ethiopia, 2019

Variables	Categories	Frequency(*n* = 384)	Percent (%)
Sex	Male	146	38
	Female	238	62
Age categories	< 20	23	6
	21–30	100	26
	31–40	92	24
	41–50	92	24
	> 51	77	20
Occupation	Farmer	192	50
	Housewife	123	32
	Merchant	8	2
	Governmental employee	15	4
	Students	6	2
	unemployed	40	10
Educational status	Illiterate	215	56
	Grade 1 to 8	145	38
	High school and above	24	6
Family size	1–2	38	10
	3–5	200	52
	> 6	146	38
Walking distance home to health facility in minute	< 60 min	77	20
	> 60 min	307	80

### Knowledge and practice of participants on cutaneous leishmaniasis

In the present study, 382 (99%) of the study participants replied that they had heard of CL. Two hundred ninety-eight (78%) of the participants stated that CL is a health problem. On the other hand, 168 (44%) of the interviewees did not know that CL could be cured with modern treatment. Of the household heads (99%) replied that lesion on the face (nose, ear, forehead, and lip) is the most common clinical presentation of CL. In addition, most of the study participants (82%) stated that CL is a preventable disease. However, a majority of the participants remain unable to identify the most common prevention method for CL such as used of a bed net (0%), and chemical house spray (0%). A large number of the study household heads stated personal hygiene (46%), and environmental sanitation (44%) were among the most common CL prevention methods. In general based on the total scoring result, about 323 (84%) of the study participants were knowledgeable for CL (Table 2).

**Table 2:** Knowledge and practice of study participants on cutaneous leishmaniasis in Ganta-afeshum, Tigrai, northern Ethiopia, 2019

Questions	Categories	Frequency (%)
**Participants knowledge on cutaneous leishmaniasis**		
Have you ever heard about cutaneous leishmaniasis? (*n* = 384)	Yes	382 (99.5)
	No	2 (0.5)
Do you believe cutaneous leishmaniasis is a health problem? (*n* = 382)	Yes	313 (82)
	No	69 (18)
Do you believe cutaneous leishmaniasis can be cured through treatment? (*n* = 382)	Yes	8 (2)
	No	206 (54)
	Don’t know	168 (44)
Mode of cutaneous leishmaniasis transmission? (*n* = 382)	Sand fly	0 (0)
	Contact with infected lesion	42 (11)
	Poor personal hygiene	118 (31)
	Climate change	148 (39)
	Genetically	75 (19)
What are the sign and symptom cutaneous leishmaniasis? (*n* = 382)	Lesion on face(cheek, chin, nose, ear, forehead, lip)	380 (99.5)
	Itching skin	154 (40)
	Fever	15 (4)
Location of lesion or scar? (*n* = 382)	Face (cheek nose, ear, forehead, chin, lip)	380 (99.5%)
	Hand	20 (5)
	Leg	4 (1)
Have you ever heard about the sand fly? (*n* = 384)	Yes	0 (0)
	No	384 (100)
Do you believe cutaneous leishmaniasis can be prevented? (*n* = 382)	Yes	313 (82)
	No	69 (18)
If “yes” what are the prevention measures of CL? ***n*** **= 313**	Using bed net	0 (0)
	House spray with chemical	0 (0)
	Personal hygiene	145 (46)
	Environmental sanitation	137 (44)
	Isolating from infected person	31 (10)
Overall knowledge	Good	323 (84)
	Poor	61 (16)
**Participants practice on preventive measure of cutaneous leishmaniasis**		
Health education for CL	Yes	0 (0)
	No	384 (100)
Sleeping under nets	Yes	0 (0%)
	No	384 (100)
House spray with chemical on last 12 month	Yes	0 (0)
	No	384 (100)
Control and fill cracks and animal borrow	Yes	106 (28)
	No	278 (72)
Working outsides during the night?	Yes	361 (94)
	No	23 (6)
Do you sleep outdoor during the night?	Yes	238 (62)
	No	146 (38)
If “yes” Sleeping conditions? ***n*** **= 238**	Near agricultural and vegetation area	22 (9)
	Near animal house	216 (91)
Overall practice status	Good	69 (18)
	Poor	315 (82)

Regarding the practice toward preventative measures of CL, all of the study participants 384 (100%) stated that they had never been given health education about CL. The household heads also stated that they were not sleep under bed nets during night. In addition, all of the participants claimed that their house was not sprayed in the last year before data collection. Majority 94% (361/384) of the study participants practiced outdoor working activities during the night. Nearly 62% (238/384) of respondents sleep outdoors, most commonly close to animals. According to the requested questions, 82% (315/384) of the study household heads scored less than the average mean. Overall the practice level of the households toward CL was poor (Table 2).

Table 3 summarizes the overall relationship between the demographic variable and the knowledge, and practice status of the participants. Among the total participants, 200 females and 122 males had good knowledge toward cutaneous leishmaniasis. However, there were no statistically significant associations (*p* = 0.964) between the sex and knowledge status of participants. Similarly, no statistical difference (*p* = 0.349) was observed between educational status of respondents and knowledge on the disease. On the other hand, age categories (*p* = 0.000) and occupation (*p* = 0.000) of the participants were significantly associated with knowledge of the diseases. In this study, a majority of the male (123/146) and female (192/238) participants poorly practiced preventative measures for CL. This study found a significant association between the demographic variables such as: age categories (*p* = 0.000), occupation (*p* = 0.000), household family size (*p* = 0.000) and practice status of respondents on cutaneous leishmaniasis.

**Table 3:** Chi-square test analysis between demographic variables and knowledge, practice status of CL among residence of Ganta-afeshum district, Tigrai, northern Ethiopia, 2019

Variable	Categories	Knowledge status		X^2^	*P*-value	Practice status		X^2^	*P*-value
		Good	Poor			Good	Poor		
Sex	Male	122	24	0.002	0.964	23	123	0.800	0.371
	Female	200	38			46	192		
Age categories	< 20	20	3	61.070	0.000^*^	2	21	65.964	0.000*
	21–30	62	38			23	77		
	31–40	91	1			38	54		
	41–50	76	16			2	90		
	> 51	69	8			7	70		
Occupation	Farmer	169	23	12.238	0.032*	38	154	22.900	0.000*
	Housewife	94	29			22	101		
	Merchant	6	2			3	5		
	Governmental employee	12	3			7	8		
	Student	4	2			3	3		
	Unemployed	31	9			5	35		
Educational status	Illiterate	178	37	3.287	0.349	36	179	5.208	0.157
	Grade 1 to 8	127	18			23	122		
	High school and above	21	3			5	16		
Family size	1–2	31	7	.620	.734	7	31	24.492	0.000*
	3–5	169	31			52	148		
	> 6	122	23			8	137		
Distance home to health facility	< 60 min	62	15	1.152	.283	37	40	67.435	0.000*
	> 60 min	263	47			30	280		

* = *p*-value < 0.05 (statistically significant association)

### History and treatment-seeking behavior on cutaneous leishmaniasis

The history and treatment-seeking behavior of participants on CL is summarized in Table 4. One hundred forty six (38%) of the study participants responded that at least one member of the household had been previously infected with CL. A maximum of three CL cases were reported in some of the households. A majority of the respondents stated females were more affected by CL than males. Children less than 5 years old were reported as most affected by CL (45%, 66/146), followed by children aged 6–18 years old (38%, 58/146). It was also observed that a majority of the study participants did not use modern treatment as 90% (132/146) of household members with a history of CL were treated by traditional healers.

**Table 4:** Study participant history and treatment seeking behavior of cutaneous leishmaniasis in Ganta-afeshum, Tigrai, and northern Ethiopia

Questions	Categories	Frequency (%)
Household with history of cutaneous leishmaniasis	Yes	146 (38)
	No	238 (62)
Most cutaneous leishmaniasis affected groups? ***n*** **= 146**	< 5 years children	66 (45)
	6–18	58 (38)
	> 19	22 (17)
Gender affected ***n*** **= 146**	Male	44 (30)
	Female	102 (70)
Did you seek treatment for the cutaneous leishmaniasis from any source? ***n*** **= 146**	Yes	146 (100)
If “Yes” Where was taken treatment?	Health facilities	14 (10)
	Traditional healer	132 (90)

## Discussion

This study indicated that nearly all (99%) of household heads had heard of CL, which is comparable to the current literature [21, 24]. In addition, most of the respondents (78%) realized that CL is a health problem in the study area. A high knowledge level of CL in the community may be due to the endemic nature of the disease in the area [6, 25].

The present work revealed that all of the study participants did not know the mode of CL transmission. This finding is supported by studies conducted in Pakistan [22] and Saudi Arabia [26]. About 31, 39 and 19% of the study participants revealed that CL is transmitted by poor personal hygiene, climate change and genetically, respectively. Taken all together, the poor knowledge of the participants on the mode of CL transmission might be due to the low educational level and lack of health education in the area, as more than half of the respondents in this study were illiterate. Moreover, almost all the participants claimed that they had never received adequate health education about the disease. In contrast, a study conducted in Isfahan, Iran reported that 97.9% of study participants had good knowledge on the transmission of CL by sand flies [27]. This difference may be attributed to the high educational level in Iranian study participants.

Regarding clinical presentation, almost all of the study participants mentioned skin lesions, primarily on the face, as the main symptom of CL. Other previous studies have revealed similar findings [28–30]. A previous report suggested that more than half of CL lesions were located on the face [31]. This may be because the face is readily exposed to sand flies. Most of the study participants incorrectly believed that CL cannot be cured by modern treatments. The absence of CL diagnosis and treatment centers in the area might influence the awareness of the community on modern treatment. Moreover, as most individuals live in rural and remote areas with limited access to transportation, traditional medicine may be preferred [32].

People living in CL endemic areas must be aware of sand fly resting, breeding sites and feeding behaviors to minimize exposure [33]. However, in the present study all of the respondents have never heard of sand flies. Moreover, the study participants were unable to differentiate sand flies from mosquitoes and other biting flies. In other studies, fairly good awareness on sand flies behavior were reported from Pakistan (20%) [22], south Iran (18%) [34] and Isfahan, Iran (13.9%) [35]. A study from Ecuador revealed high knowledge (82%) of participants on sand fly characteristics [23]. The possible explanation for this difference might be due to difference in awareness, the inability to identify the sand flies, and the endemicity of the area.

With regard to prevention of CL, about 82% of the study participants believed that CL is a preventable disease. On the contrary, a study in Saudi Arabia reported only 19.3% of the participants assumed that the disease is preventable [26]. The high awareness of CL in this study is attributable to its endemicity in the area. However, most of the participants misunderstood the main prevention and control methods of CL; no one is mentioning the use of bed nets, and chemical house spraying as prevention methods. Moreover, most of the respondents mentioned environmental sanitation (44%), personal hygiene (46%) and isolation from an infected person (10%) as the main CL preventing methods. This finding was similar to that of a study in Ochello, South Ethiopia [36].

Regarding the practice of CL preventative measures, almost all of our study participants (100%) had stated that no health education was given in the area. Similarly, all the participants claimed that they had not any ITNs and their house had not yet been sprayed up to 1 year prior to data collection. Poor CL control practice may suggest an absence of strong CL prevention and control effort in the country. Nationwide, the national leishmaniasis prevention strategy was established late in 2007, with CL only incorporated in 2013 [16]. It is believed that the distribution of insecticide-treated net and house spraying to prevent malaria have a positive impact on the control of sand flies [37]. However, there has been limited effort to distribute insecticide-treated nets and house spraying in the study area as it is not endemic for malaria.

In the present study, 38% of the study participants stated that at least one member of the household had been infected with CL. In Ethiopia, the prevalence of CL ranging from 14 to 65.4% has been reported [24, 30]. In this present study, female had a higher rate of previous CL lesion. It is noticeable that the distribution of CL is influenced by gender-specific factors like social habits, gender inequality due to preferential treatment in rural communities, lack of knowledge and occupational roles toward CL, and the social stigma associated with the disease that often keeps females hidden [38]. Although CL affects individuals of any age, 45% of the study participants with history of CL were children less than 5 years old while 38% were aged between 6 and 18 years old. This finding is in agreement with many studies in Ethiopia [39–41]. Children, particularly females, were found to be most exposed to sand fly bites, as they usually have more active roles in farming activities, taking care of cattle, and handling manure [42].

Most of the respondents (90%) with history of CL lesion are treated by traditional medicine. This may be attributed to the poor practice of modern CL treatment in the study area. There is only one specialized hospital in Tigrai region that diagnoses and treats CL, however it is found far from the study areas. Studies from India [43] and Ghana [44] also found 100 and 75% of participants having CL were treated by traditional healer. Previous research suggests that “herbs and plants” were used as common traditional therapeutics for CL [45, 46]. This may be due to the long duration, high cost, side effects, and drug resistance attributed to modern therapeutics [47]. Although some of the study participants with a history of CL experienced herbal medicine, most of them were unable to explain what treatments were used. Hence, the efficacy of the traditional medicine might vary accordingly and the traditional healers would not publicize the treatments implemented for CL.

## Conclusion

In conclusion, a lack of awareness concerning the transmission of CL in Ganta-afeshum district was established. The findings suggest that individuals in this district are not familiar with sand-flies. As well, many participants suggested that they do not believe modern therapeutics could cure CL. These ideologies may have a negative impact on efforts to control sand flies and their animal reservoirs and may enhance transmission of the disease. The observational data suggested that preventative measures for CL, such as utilizing insecticide-treated nets and indoor residual spraying were not practiced within the community. Many participants reported sleeping outdoors, increasing exposure to sand flies significantly. In all of the instances, health education programs regarding to CL transmission, prevention, and treatment should be rigorously implemented in the area.

### Limitation of the study

The interview questions for the knowledge and practice towards treatment-seeking behavior of the households may be prone to social desirability bias. Hence, the findings of this study should be interpreted with caution. Overall, this finding can be a proxy indicator of the community knowledge, practice, and treatment-seeking behavior towards CL.

## Supplementary Information

**Additional file 1.** Questionnaires for evaluating household knowledge, practice and treatment seeking behavior toward cutaneous leishmaniasis.

## Data Availability

All data generated or analyzed during the study are included in the manuscript.

## Abbreviations

CL: Cutaneous leishmaniasis
ITNs: Insecticide-treated nets
IRS: Indoor residual spray
