# Supplementary material for: Household knowledge, practice and treatment seeking behaviors towards cutaneous leishmaniasis in the endemic rural communities of Ganta- afeshum district, Tigrai, northern Ethiopia, 2019: a cross-sectional study
Source: Trop Dis Travel Med Vaccines. 2021 Jun 15;7:19. doi: 10.1186/s40794-021-00144-4 (PMC8204582; doi:10.1186/s40794-021-00144-4)
Supplement: Supplementary file 1 — Additional file 1. Questionnaires for evaluating household knowledge, practice and treatment seeking behavior toward cutaneous leishmaniasis. [file 40794_2021_144_MOESM1_ESM.docx]

## Questionnaire in English

Area of research conducted: ________________________________ HH selection number_____ ________________

Date: ________________

**Part-I:** Questionnaire about socio demographic characteristics of Household in Ganta - Afeshum district

**NB; circle answer of respondents from the list**

1. Sex: A. Male B. Female
2. Age __________________
3. Residence: 1. rural 2. urban
4. Occupation :
5. Farmer
6. Housewife
7. Merchant
8. Government employee
9. Student
10. Unemployed
11. Other_____________
12. Religion of household head ;
13. Orthodox
14. Islam
15. Catholic
16. Protestant
17. other
18. Household families size:
19. 1-2
20. 3-5
21. >5
22. Distance from home to nearest health facilities in minute
23. <60 min
24. >60m
25. Educational status
26. Illiterate
27. Grade 1- 8^th^
28. Grade 10- 12^th (^high school)
29. Diploma and above
30. Type of house: NB; circle answer of respondents ○
31. Tukul with thatched roof
32. Rectangular corrugated with iron
33. Monthly income of HH
34. >150 US$(high)
35. 60-90 US$(medium)
36. >30US$(lower)

**Part-II:** Household survey for Cutaneouse leishmaniasis risk factor evaluation

1. Have you ever heard about Cutaneouse leishmaniasis? If your answer is ‘No’ Skip to Q ‘18’
2. Yes
3. No
4. Either Cutaneouse leishmaniasis in this area?
5. Ye
6. No
7. Don’t No
8. Do you believe that Cutaneouse leishmaniasis is a health problem?
9. Yes
10. No
11. Don’t know
12. Do you believe that Cutaneouse leishmaniasis can be cured through treatment?
13. Yes
14. No
15. Don’t know
16. How do you think Cutaneouse leishmaniasis is transmitted? **NB ; circle that all apply, do not prompt**
17. Sand fly bite
18. Contacted with infected persons lesion
19. Poor hygiene
20. Biting any flies
21. Environmental change (cold to hot or hot to cold)
22. Other___________________________(specify)
23. Don’t know
24. What are the symptoms of Cutaneouse leiahmainiasis? **NB: circle that all apply, without prompt**
25. Lesion on face, forehead, nose
26. Itching skin
27. Wight loss
28. Fever
29. Other ________________________
30. Location of lesion or scar
31. Forehead
32. Face
33. Nose
34. Arm
35. Leg
36. Ear
37. Mixed
38. Don’t no know
39. Have you ever heard about sand fly?
40. Yes
41. No
42. Where dose sand flies breed?
43. Vegetation
44. House crack
45. Animal dung
46. Others _______________________
47. Don’t know
48. Do you believe Cutaneouse leishmaniasis can be prevented? If your answer is ‘No or Don’t know’ skip to Q ‘23’
49. Yes
50. No
51. Don’t know
52. How can you prevent yourself from getting Cutaneouse leishmaniasis? **NB; circle all that apply do not prompt**
53. Using of bed net
54. Spraying the house with DDT
55. Isolating infected person
56. Personal hygiene
57. Cleaning dump, animal dung and vegetation area
58. No prevention method
59. Have you ever used one of the activities you just mentioned for CL prevention?
60. Yes.
61. No
62. Do you sleep outdoor during night
63. Yes
64. No
65. Sleeping conditions
66. Near vegetation under bed net
67. Near vegetation without under bed net
68. Near animal dung with bed net
69. Near animal dung without bed net
70. Never sleep near vegetations and animal dung
71. Working outsides during night
72. Yes
73. No
74. Control and fill cracks and animal borrow
75. Yes
76. No
77. Has anyone in your household been ill with Cutaneouse leishmaniasis in the last 12 month? If your answer is’ No or Don’t know skip to Q ’33’
78. Yes
79. No
80. Don’t know
81. How many members of your household were ill with Cutaneouse leishmaniasis in the last 12 months? Number ______________
82. Who was ill
83. Children under five years, sex_________ age___________
84. Adolescence , sex__________ , age ___________
85. Adult , sex __________, age_____________
86. Mother, age_______________
87. Father, age_________________
88. Did you seek treatment for the Cutaneouse leishmaniasis from any source? If your answer is ‘No’ skip to Q ‘30’
89. Yes
90. No
91. Don’t know
92. Where was taken for treatments?
93. From governmental health facility
94. Traditional healer
95. Others______________________
96. How quick was take for treatment?
97. Within two - four weeks
98. After several months
99. After years
100. Don’t’ know
101. What was the main reason for not taking for treatment? **Circle all that apply, do not prompt**
102. Don’t think CL is cures through treatment
103. Health facilities are not available in the area
104. There was no one to take Him /Her for treatment
105. I don’t like it
106. Other____________________
107. Do you think sleeping under the bed net protect from Cutaneouse leishmaniasis disease?
108. Yes
109. No
110. Don’t know
111. Does your household have any bed nets that can be used while sleeping? If your answer is ‘No ‘skip to Q ‘46’ IF Don’t know skip to Q’47’
112. Yes
113. No
114. Don’t know
115. Where do you get the bed net?
116. Gov, health facility
117. Private market
118. Other _________________
119. What is the main reason for not having a mosquito net in your household?
120. Bed nets are not available in the area
121. The cost is too high
122. Don’t think it prevent Cutaneouse leishmaniasis at all
123. We don’t like it
124. Other ____________________
125. Do you think that house and environmental spraying with chemical prevents Cutaneouse leishmaniasis?
126. Yes
127. No
128. Don’t know
129. Was your house and environment sprayed with chemicals to protect your family from getting Cutaneouse leishmaniasis in the last 12 months?
130. Yes
131. No
132. Don’t know
133. What was the importance of the spray and what does it protect?
134. From mosquito bite
135. From any sand fly
136. From house bugs bite
137. Don’t know
